# Anti-Inflammatory Effect ofEmodin via Attenuation of NLRP3 Inflammasome Activation

**DOI:** 10.3390/ijms16048102

**Published:** 2015-04-10

**Authors:** Ji-Won Han, Do-Wan Shim, Woo-Young Shin, Kang-Hyuk Heo, Su-Bin Kwak, Eun-Jeong Sim, Jae-Hyun Jeong, Tae-Bong Kang, Kwang-Ho Lee

**Affiliations:** 1 Department of Biotechnology, College of Biomedical & Health Science, Research Institute of Inflammatory Diseases, Konkuk University, Chungju 380-701, Korea; E-Mails: frbgf@naver.com (J.-W.H.); simdw871113@gmail.com (D.-W.S.); wooyoung031@naver.com (W.-Y.S.); finsth@hanmail.net (K.-H.H.); subini1227@naver.com(S.-B.K.); sim1023@hanmail.net (E.-J.S.); kangtbko@gmail.com (T.-B.K.); 2 Department of Food Science & Technology, Korea National University of Transportation, Chungju 368-701, Korea; E-Mail: jhjeong@cjnu.ac.kr

**Keywords:** emodin, interleukin (IL)-1β, NLRP3 inflammasome, sepsis

## Abstract

Emodin, an active constituent of oriental herbs, is widely used to treat allergy, inflammation, and other symptoms. This study provides the scientific basis for the anti-inflammasome effects of emodin on both *in vitro* and *in vivo* experimental models. Bone marrow-derived macrophages were used to study the effects of emodin on inflammasome activation by using inflammasome inducers such as ATP, nigericin, and silica crystals. The lipopolysaccharide (LPS)-induced endotoxin shock model was employed to study the effect of emodin on *in vivo* efficacy. Emodin treatment attenuated interleukin (IL)-1β secretion via the inhibition of NOD-like receptor family, pyrin domain containing 3 (NLRP3) inflammasome activation induced by ATP, nigericin, and silica crystals. Further, emodin ameliorated the severity of NLRP3 inflammasome-mediated symptoms in LPS-induced endotoxin mouse models. This study is the first to reveal mechanism-based evidence, especially with respect to regulation of inflammasome activation, substantiating traditional claims of emodin in the treatment of inflammation-related disorders.

## 1. Introduction

Interleukin (IL)-1β has a central role in the inflammatory process by inducing expression of adhesion molecules and cell recruitment into the site of infection. Inflammasomes are critical for activation of this proinflammatory interleukin-1 cytokine family [1]. The NOD-like receptor family, pyrin domain containing 3 (NLRP3) inflammasome is the most widely studied among the inflammasomes, and known to be activated upon signs of cellular “danger signals”. The key components of the functional NLRP3 inflammasome are NLRP3, the adaptor protein apoptosis associated speck like protein (ASC), and caspase-1 [2]. Activating stimuli for the NLRP3 inflammasome are diverse and include both endogenous factors (ATP, uric acid crystals, *etc*.) and exogenous factors (bacterial hemolysins, pneumolysin, *vs*.). NLRP3 inflammasome is implicated in diseases, such as Alzheimer’s disese [3], obesity [4], major depressive disorder [5], obesity induced type 2 diabetes [6], and fibromyalgia [7]. Therefore, suppressing the cleaved IL-1β production through the modulation of NLRP3 inflammasome activation should be an important strategy for relieving inflammation-related disorders.

Emodin (1,3,8-trihydroxy-6-methylemodin) is an active constituent of oriental medicinal herbs including *Rheum officinale* and *Polygonum cuspidatum* [8]. Reports have demonstrated that emodin possesses biological functions such as anti-bacterial [9], vasorelaxant [10], anti-cancer [11], and anti-allergy [12,13]. Many studies related to the anti-inflammatory potential of emodin also have been reported [14,15,16]. However, most of these anti-inflammation-related studies have been examined with LPS/Toll like recector (TLR)s regulation, but the functional involvement of emodin in IL-1β secretion through regulation of inflammasome has hitherto not been reported. In the present investigation, we demonstrate the functional capacity of emodin in improvement of inflammation through regulation of inflammasomes and IL-1β secretion.

## 2. Results and Discussion

### 2.1. Effects of Emodin on Inflammasome-Induced Interleukin (IL)-1β Secretion in Vitro

**Figure 1:**
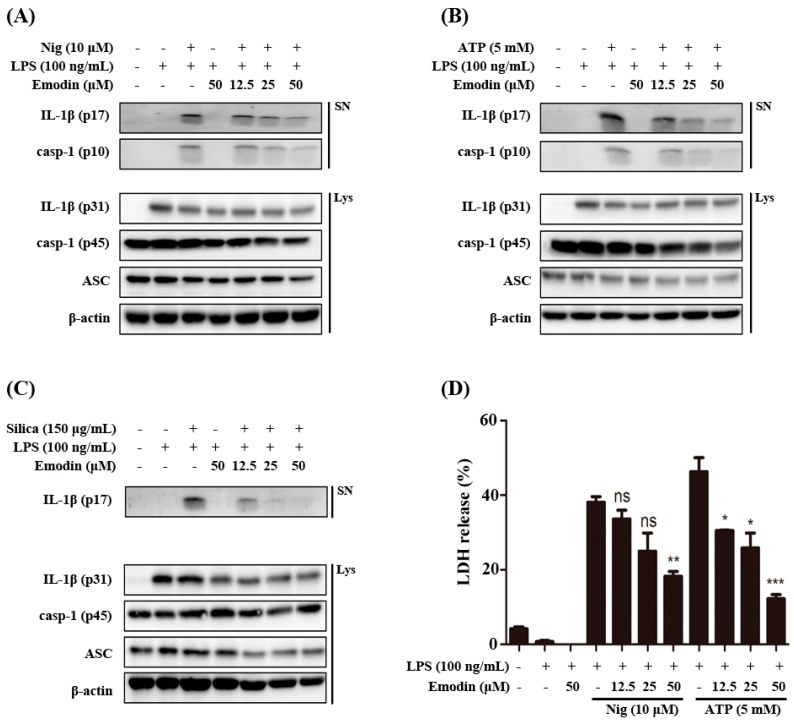
Effects of emodin on inflammasome-induced interleukin (IL)-1β secretion and cell death *in vitro.* LPS-primed bone marrow-derived macrophages (BMDMs) were pretreated with emodin for 1 h and stimulated with (**A**) nigericin (Nig) (10 μM) and (**B**) ATP (5 mM) for 1 h, and (**C**) silica crystals (silica) (150 μg/mL) for 3 h, and supernatants (SN) were used to detect cleaved IL-1β (p17) and caspase-1 (p10). Cell lysates (Lys) were used to detect internal controls, such as pro-IL-1β (p31), ASC, pro-caspase-1 (p45), and β-actin; (**D**) LPS-primed BMDMs were treated with emodin at the indicated concentration for 1 h and then treated with nigericin (Nig) (10 μM) and ATP (5 mM) for 1 h, after which supernatants were collected for LDH determination. The results are expressed as mean ± S.E.M. of three independent experiments in triplicate. * *p* < 0.05, ** *p* < 0.01 and *** *p* < 0.001 compared with Nig, ATP treated cells. ns: None significant.

Pro-IL-1β is known to be induced by pro-inflammatory signals such as LPS or tumor necrosis factor (TNF) that activate the NF-κB transcription factors, and the release of bioactive IL-1β from macrophages is in turn dependent on activation by inflammasome. The NLRP3 inflammasome has been widely studied and responds to a broad range of pathogen-associated molecular patterns (PAMP) or damage-associated molecular patterns (DAMP) signals such as monosodium urate crystal (MSU), ATP, and nigericin which are very well known NLRP3 inflammasome inducers. We investigated the potency of emodin on the regulatory effect on NLRP3 inflammasome activation. As shown in Figure 1A–C, treatment with emodin inhibited the secretion of cleaved IL-1β and caspase-1 in culture supernatants from bone marrow-derived macrophages (BMDMs) responding to danger signals such as nigericin, ATP, and silica crystals. However, other inflammasome components have not been changed in cell lysates.

Activated inflammasomes serve as a platform for caspase-1 activation and IL-1β maturation, as well as pyroptosis [17]. We determined the effects of emodin on pyroptosis caused by cell death. As shown in Figure 1D, emodin inhibited the inflammasome induced cell death dose dependently. Results indicate that emodin can regulate NLRP3 inflammasome activation, leading to attenuation of IL-1β and caspase-1 secretion.

### 2.2. Effects of Emodin on Inflammasome Component and Sepsis in Vivo Model

**Figure 2:**
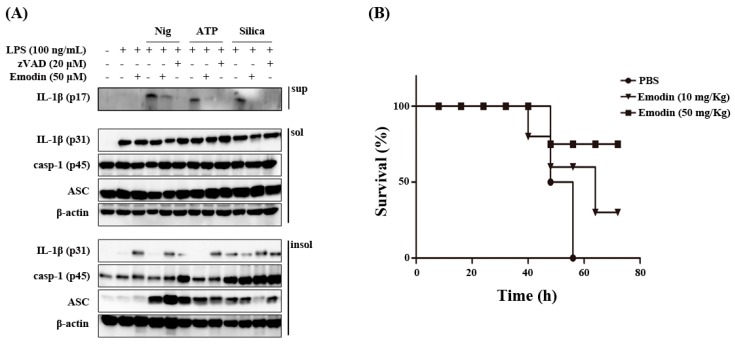
Effects of emodin on inflammasome component *in vitro* and sepsis *in vivo* model. (**A**) LPS-primed BMDMs were pretreated with emodin for 1 h and stimulated with nigericin (Nig) (10 μM) and ATP (5 mM) for 1 h, and silica crystals (Silica) (150 μg/mL) for 3 h, and supernatants were used to detect cleaved IL-1β. Protein levels of NLRP3 inflammasome components in Triton X-100 soluble (sol) and Triton X-100 insoluble (insol) fraction lysates were used to detect internal controls, such as pro-IL-1β (p31), ASC, pro-caspase-1 (p45), and β-actin; (**B**) Mice (*n* = 5 per each group) were intraperitoneally treated with LPS (20 mg/kg) with or without emodin (10 and 50 mg/kg) and LPS (20 mg/kg). Survival rates were observed at indicated times.

It has been reported that inflammasomes are large multiprotein oligomers containing mainly the adaptor protein apoptosis-associated specklike protein (ASC), caspase-1, and NLRP3 [18]. The ASC forms oligomers associating with mitochondria-associated ER membranes MAMs [19]. ASC also have been reported to form Triton X-100 resistant aggregates [20,21]. Accordingly, we examined inflammasome components in Triton X-100 soluble and insoluble fraction. As shown in Figure 2A, emodin significantly inhibited cleaved IL-1β production in the supernatant. However, emodin did not affect inflammasome-related protein expression in Triton X-100 soluble fraction. Interestingly, emodin elevated pro-IL-1β level in Triton X-100 insoluble fraction. It remains to be elucidated how emodin halts pro-IL-1β in the insoluble fraction, leading to inhibition of caspase-1 activity.

LPS-induced endotoxin shock is reduced in NLRP3 gene-deleted mice compared to intact mice [22]. Thus, we confirmed the inhibitory effect of emodin on NLRP3 inflammasome activation by the LPS-induced endotoxin shock model. As shown in Figure 2B, mice intra peritoneally treated with emodin showed higher survival rates than LPS alone injected control mice, indicating that emodin can ameliorate the severity of NLRP3 inflammasome-mediated disease symptoms *in vivo*.

Taken together, our study showed that emodin inhibited NLRP3 inflammasome activation *in vitro* and LPS-induced endotoxin shock in *in vivo* model. The potential effects of emodin in inflammasome derived inflammatory responses might provide a valuable therapeutic strategy in controlling inflammation-mediated pathological disorders.

## 3. Experimental Section

### 3.1. Reagents and Antibodies

Penicillin-streptomycin, fetal bovine serum, and RPMI 1640 were purchased from Gibco (Grans Island, NY, USA). A 3,3',5,5'-tetramethylbenzidine (TMB) substrate reagent set was purchased from BD Biosciences (Franklin Lakes, SD, USA). IL-1β antibody was purchased from R&D systems (Minneapolis, MN, USA). ASC and NLRP3 antibodies were purchased Enzo Life Sciences (Farmingdale, NY, USA). Caspase-1 and β-actin were purchased from Santa Cruz Biotechnology (Santa Cruz, CA, USA). Silica crystal, nigericin, and zVAD-FMK were purchased from InvivoGen (San Diego, CA, USA). A Western blot chemiluminescence reagent kit (Super Signal West Pico Stable Peroxide and Super Signal West Pico Luminol/Enhancer solutions) was purchased from Pierce Chemical (Rockford, IL, USA). Polyvinylidene fluoride (PVDF) membrane was purchased from Millipore Corporation (Bedford, MA, USA). LPS (*E. coli* 026:B6), ATP, and emodin were purchased from Sigma-Aldrich (St. Louis, MO, USA). A LDH kit was purchased from BioVision (Mountain View, CA, USA).

### 3.2. Preparation and Culture of Bone Marrow-Derived Macrophages (BMDMs)

Bone marrow cells from C57BL/6 mice (8 weeks old) were collected by flushing the femurs and tibias in sterile PBS. The collected cells were cultured in RPMI 1640 supplemented with 10% fetal bovine serum (FBS), 30% L929 cell-conditioned medium (LCM), and 1% penicillin-streptomycin for 7 days to differentiate into macrophages (BMDMs). Cells were seeded in non-tissue culture-treated Petri dishes (SPL Life Science Co., Gyeonggi-do, Korea) and incubated at 37 °C in a 5% CO_2_ atmosphere.

### 3.3. Animals

All experiments were performed in accordance with the guidelines of the Konkuk University Animal Care Committee, Korea. Male C57BL/6 mice (20–22 g, 6 weeks old) were purchased from Orient Bio Co., Korea. They were housed in groups of five under standard conditions (temperature 22% ± 2 °C, humidity 55% ± 5%, 12 h light/dark cycle) with food and water *ad libitum*. The animals were allowed to adapt to the laboratory environment for 5 to 7 days before experimentation. The experiments were conducted in accordance with the “Guide for the Care and Use of Laboratory Animals” adopted by the United States National Institutes of Health.

For LPS-induced endotoxin shock, mice were intraperitoneally co-injected with LPS plus vehicle (200 μL of saline) or emodin. The animals were observed every 8 h for 3 days.

### 3.4. Inflammasome Activation

BMDMs (1.0 × 10^6^ cells/well) were plated in 6-well plates and primed with LPS (100 ng/mL) for 3 h. After LPS priming, the medium was replaced with opti-MEM, and cells were incubated for 1 h with or without emodin or zVAD, before being pulsed with ATP (5 mM) and nigericin (10 µM) for 1 h and silica crystal (150 µg/mL) for 3 h.

### 3.5. Immunoblot Analysis

Immunoblot analysis was performed as described previously [23]. The experimental process of Triton X-100 soluble and insoluble fractions was previously described [17].

### 3.6. Statistical Analysis

The results were expressed as the mean ± S.E.M. of at least three independent experiments performed in triplicate. Statistical analysis was performed using the one-way ANOVA, and *p* value less than 0.05 was considered to be statistically significant

## 4. Conclusions

Emodin attenuated NLRP3 inflammasome activation, leading to decreased secretion of cleaved IL-1β and blocking of the inflammasome-induced pyroptosis. *In vivo* efficacy of the anti-inflammatory effect through regulation of inflammasome activation was verified by the sepsis mouse model. Although the detailed mechanisms that link the common molecular pathways to the means by which emodin attenuates NLRP3 inflammasome activation remains to be elucidated, this is the first report providing scientific evidence substantiating the use of emodin in medicine for the treatment of various inflammatory diseases through the regulation of inflammasome activation, which may provide a valuable therapeutic strategy for controlling inflammasome-mediated pathological conditions.
